# Quantitative measurement of hair diameter diversity as a diagnostic indicator of androgenetic alopecia in Korean males: A cross-sectional study

**DOI:** 10.1016/j.jdin.2024.02.005

**Published:** 2024-02-15

**Authors:** Hee Ung Park, Kyung Bae Chung, Do-Young Kim

**Affiliations:** a Department of Dermatology, Hanyang University Medical Center, Myongji Hospital, Goyang, Republic of Korea; b Department of Dermatology, Cutaneous Biology Research Institute, Yonsei University College of Medicine, Seoul, Republic of Korea

**Keywords:** androgenetic alopecia, diagnostic criteria, hair thickness, Korean, phototrichogram, threshold

## Abstract

**Background:**

The conventional 20% threshold for hair diameter diversity (HDD), widely accepted for diagnosing androgenetic alopecia (AGA) in the vertex area, has not quantitatively analyzed.

**Objective:**

To validate the HDD 20% threshold for AGA and develop a refined, Korean-specific criterion.

**Methods:**

This study involved 240 male patients with AGA, categorized by the V stages of the basic and specific classification. Phototrichogram images of the vertex region were analyzed using Image J software for hair thickness measurement.

**Results:**

Receiver operating characteristic curve analysis determined the 45 μm hair diameter threshold as the most diagnostic for AGA, with an area under the curve value of 0.884 and a Youden index of 0.659. Optimal AGA diagnosis was achieved when over 21% of hair had a diameter of ≤45 μm.

**Limitations:**

Restriction to Korean male limits its applicability to a broader population, and using a specific hair diameter threshold does not account for individual variations in hair characteristics.

**Conclusion:**

The study validates the conventional HDD 20% threshold and proposes a more appropriate 45 μm threshold for Korean males, beyond the 40 μm. It concludes that while the HDD 20% remains a key method for early detection of vertex AGA, the definition of thin hair should be ethnicity-specific.

**Capsule Summary**

• This study statistically validates the hair diameter diversity 20% threshold as a diagnostic criterion for androgenetic alopecia using a phototrichogram.
• Clinically, the hair diameter diversity 20% threshold is a validated method for diagnosis of androgenetic alopecia, but it should be adjusted for ethnic variations.

## Introduction

Androgenetic alopecia (AGA), the predominant form of baldness often referred to as pattern hair loss, causes individuals to commonly exhibit an abundance of thin hairs.^1^, ^2^, ^3^, ^4^, ^5^ Traditionally, AGA diagnosis has not relied on precise and objective measurements, instead, clinicians have commonly relied on subjective judgment using severity classification tools such as the Norwood-Hamilton, Ludwig, or the basic and specific (BASP) classification.^2^ Introduced in 2001, the concept of increased hair diameter diversity (HDD) is recognized as a critical objective clinical sign in diagnosing AGA, particularly due to its association with hair follicle miniaturization.^6^ Since hair follicle miniaturization stands central to AGA, HDD serves as a pivotal and easily understandable indicator of this phenomenon.^6^ Therefore, HDD, also termed anisotrichosis, is perceived in current literature as a precise marker for AGA when the diversity exceeds a threshold of 20%.^7^, ^8^, ^9^, ^10^, ^11^, ^12^ However, even in the study that introduced the HDD, the specific criteria for this HDD 20% threshold were not clearly defined.^6^ Consequently, most studies have subjectively used the proportion of thin hairs exceeding 20% as a diagnostic marker for AGA.^9^^,^^13^ While the definition of 'thin hair' varies across studies, based on the criteria set in the study that introduced HDD, hairs with a diameter of 40 μm or less can be inferred.^6^ Thus, an HDD ≥20% can be interpreted as having 20% or more hairs measuring 40 μm or less in diameter. However, since the study was based on Caucasians, the 40-μm criterion may not be appropriate for Asians, who typically have thicker hair compared to Caucasians.^14^, ^15^, ^16^, ^17^, ^18^, ^19^ Therefore, our study aims to reevaluate the HDD ≥20% criteria through quantitative measurement of hair thickness and, moreover, to refine the criteria to be more appropriate for Koreans.

## Materials and method

### Patients and study design

A total of 240 Korean male patients who visited the Department of Dermatology at Severance Hospital in Seoul, Republic of Korea, with AGA from January 2020 to August 2023, were enrolled in this study. We calculated the sample size based on an expected area under the curve (AUC) of 0.7 for HDD, intending to ensure 90% power with a type I error of 0.05 and a type II error of 0.10, which led to a prospective enrollment of 124 patients considering a dropout rate of about 10%, and a replication cohort of 116 retrospective patients. This selection was grounded in HDD's prevalent clinical use and moderate diagnostic power, despite the scarcity of literature on its exact diagnostic rate. This dual approach was utilized to increase statistical power and to validate diagnostic variations in real-world practice settings (Supplementary Figure 1, available via Mendeley at https://data.mendeley.com/datasets/f2zvbwjn3b/1). Inclusion criteria specified patients aged 18 or older with AGA who were otherwise healthy. Patients with severe underlying systemic diseases were excluded. Additionally, patients presenting combined specific hair disorders (eg, scarring alopecia, alopecia areata, telogen effluvium, and psoriasis) or abnormalities on the scalp observed during physical examinations (eg, burns or inflammation) or chart review were excluded from the study. All procedures and methodologies were performed in accordance with the relevant guidelines and regulations.

### Clinical assessment and photography

Informed consent was obtained from each participant in the prospective study, along with a detailed medical history and a clinical assessment. For the retrospective cohort, standard global photography and medical records were utilized. The photography process was standardized for all participants, using a headrest for consistent positioning and ensuring clarity in the images. Clinical photographs of the patients were classified according to the BASP classification by 2 board-certified dermatologists to precisely assess the degree of hair loss in the vertex area. Finally, patients were categorized based on the V stages of the BASP classification, with V0 serving as the control group, indicating no AGA in the vertex area.

### Phototrichogram and hair measurement

To obtain magnified images of vertex hair, a phototrichogram (Folliscope 5.0; Lead M) was utilized. Images were taken at magnifications ranging from 50x to 85x, centered on the V point (intersection between the mid-sagittal line and the coronal line connecting both tips of the tragus, 1-1.3 cm from the headband). To ensure clarity and precision, 2 to 4 vertex photographs were taken, and the clearest image without overlapping hairs was selected for further analysis. While the Folliscope 5.0 is capable of measuring hair thickness, its accuracy was found to be insufficient for the detailed requirements of this study. Therefore, for enhanced precision in hair thickness measurement, the selected image was exported from Folliscope 5.0 and analyzed at the pixel level using the Image J software (Rasband, W.S., Image J, U.S. National Institutes of Health). Calibration was conducted using a 1-mm reference captured with the Folliscope 5.0, analyzed in Image J. The diameter of all visible hairs within the image was measured, with the standard for diameter measurement set at a height of 350 to 450 μm from the point of emergence of the hair from the scalp (Supplementary Figure 2, available via Mendeley at https://data.mendeley.com/datasets/f2zvbwjn3b/1). In each case, between 16 and 75 hairs were measured, and hair density was calculated as the total number of hairs per 1-cm^2^ area. The clinical information and reference standard results were not accessible to the performers conducting the measurement.

### Statistical analysis

All statistical analyses were performed with SPSS Statistics software (for Windows, Version 29.0.0.0.IBM Corp; 2022) after the completion of clinical assessment and hair measurement. Continuous variables such as age and hair thickness were presented as means and SDs, while categorical data were expressed as frequencies and percentages. To evaluate the diagnostic accuracy of the HDD ≥20% criterion, the study explored various hair diameter thresholds for defining 'thin hair' to establish the most appropriate standard for diagnosis. Positive predictive value (PPV) and negative predictive value were analyzed, differentiating between V0 (normal) and V1 to V3 (AGA) stages. The hair diameter threshold demonstrating the highest Youden index at the 20% cutoff was identified and subjected to more detailed analysis. This involved receiver operating characteristic curve analysis for specific HDD thresholds, with the selection of the threshold providing the highest diagnostic value based on the Youden index and the cutoff percentage. There were no instances of indeterminate results or missing data.

## Result

The study enrolled 240 male patients diagnosed with AGA, classified into 4 groups based on the V stages of the BASP classification: V0 (*n* = 53), V1 (*n* = 104), V2 (*n* = 55), and V3 (*n* = 28). The overall mean age of the patients was 38.5 ± 12.4 years. Analyzed by group, the mean age for V0 was the lowest at 31.2 ± 8.4 years, indicating that patients with AGA without vertex involvement (control group) were younger. V1 patients had a mean age of 36.7 ± 12.2 years, V2 patients were older at 43.9 ± 11.7 years, and V3 patients, representing the most advanced stage of AGA, had the highest mean age of 48.1 ± 9.4 years. These demographic findings indicate a trend where the mean age and age distribution correlate with the severity of AGA, which is consistent with the progressive nature of AGA (Table I).

**Table I:** Baseline characteristics of male patients with androgenetic alopecia by basic and specific V stage

Characteristics	Total (*N* = 240)	BASP V stage			
		V0 (*n* = 53)	V1 (*n* = 104)	V2 (*n* = 55)	V3 (*n* = 28)
Age (y)					
Mean (±SD)	38.5 ± 12.4	31.2 ± 8.4	36.7 ± 12.2	43.9 ± 11.7	48.1 ± 9.4
10-19, *n* (%)	6 (2.5)	1 (0.4)	5 (2.1)	0 (0)	0 (0)
20-29, *n* (%)	65 (27.1)	27 (11.3)	31 (12.9)	7 (2.9)	0 (0)
30-39, *n* (%)	69 (28.7)	17 (7.1)	31 (12.9)	15 (6.3)	6 (2.5)
40-49, *n* (%)	40 (16.7)	5 (2.1)	14 (5.8)	12 (5.0)	9 (3.8)
50-59, *n* (%)	50 (20.8)	3 (1.3)	20 (8.3)	16 (6.7)	11 (4.6)
60 and above, *n* (%)	10 (4.2)	0 (0)	3 (1.3)	5 (2.1)	2 (0.8)
Duration of AGA (y)					
Mean (±SD)	7.4 ± 6.0	5.2 ± 3.9	6.1 ± 4.9	8.7 ± 6.0	12.9 ± 7.8

*AGA*, Androgenetic alopecia; *BASP*, basic and specific.

Average hair thickness decreased with the severity of AGA, with the thickest average hair measurements observed in the V0 group (51-98 μm, mean 70 ± 10 μm) and the thinnest in the V3 group (24-65 μm, mean 38 ± 8 μm). Hair density showed a marginal decrease from V0, with values ranging from 77 to 231 hair/cm^2^ (mean 149 ± 30 hair/cm^2^), to V3 where the range was 46 to 208 hair/cm^2^ (mean 122 ± 37 hair/cm^2^). The percentage of thin hair, defined in the paper that first introduced HDD as having a diameter of ≤40 μm,^6^ demonstrated a significant increase with advancing BASP V stages. In the V0 group (control), patients exhibited 0% to 35% thin hair (mean 13 ± 8%), whereas in the V3 group, representing the most advanced stage of AGA, there was a higher range of 18% to 94% thin hair (mean 62 ± 19%) (Table II).

**Table II:** Quantitative analysis of hair characteristics based on basic and specific V stage

BASP V stage	Average hair thickness (μm)		Hair density (hair/cm^2^)		Thin hair∗ (%)		Intermediate hair∗ (%)		Thick hair∗ (%)	
	Range	Mean (±SD)	Range	Mean (±SD)	Range	Mean (±SD)	Range	Mean (±SD)	Range	Mean (±SD)
V0 (*N* = 53)	51-98	70 ± 10	77-231	149 ± 30	0-35	13 ± 8	0-89	53 ± 20	4-94	34 ± 21
V1 (*N* = 104)	31-86	57 ± 10	74-200	131 ± 27	0-78	27 ± 15	20-96	58 ± 16	0-67	15 ± 15
V2 (*N* = 55)	33-75	49 ± 9	71-216	126 ± 32	6-79	38 ± 18	21-84	54 ± 15	0-41	8 ± 9
V3 (*N* = 28)	24-65	38 ± 8	46-208	122 ± 37	18-94	62 ± 19	6-70	35 ± 18	0-32	3 ± 6

*BASP*, Basic and specific.

∗ Thin hair of ≤40 μm, intermediate hair of 40 to 80 μm, thick hair of >80 μm.

When assessing the diagnostic accuracy of the HDD ≥20% criterion using PPV and negative predictive value, we observed that applying a 40 μm threshold, the initially suggested criterion for Caucasian ethnicity,^6^ results in a PPV of 73.8%. This is significantly lower than the PPVs obtained at the 45-μm threshold and 50-μm threshold, which are 86.6% and 90.4%, respectively (Table III). This suggests that utilizing a 40 μm standard may result in a higher rate of false negatives among Korean individuals. Moreover, the negative predictive value at 40 μm is 79.2%, in contrast to 73.6% at a 45-μm threshold and 54.7% at a 50-μm threshold (Table III). These findings imply that a 45-μm threshold could be more suitable for Koreans population than the 40 μm threshold, as it offers a more balanced diagnostic accuracy in distinguishing between V0 (normal) and V1 to V3 (AGA) stages.

**Table III:** Comparison of predictive values for androgenetic alopecia diagnosis based on hair diameter thresholds

Predictive value	Diagnostic accuracy (at least 20% of hairs with a diameter under HDD μm)			
	HDD 30 μm	HDD 40 μm	HDD 45 μm	HDD 50 μm
Positive predictive value∗ (%)	35.3	73.8	86.6	90.4
Negative predictive value∗ (%)	92.5	79.2	73.6	54.7

*HDD*, Hair diameter diversity.

∗ 'Positive predictive value' indicates the proportion of individuals with AGA (V1-V3) who have at least 20% of their hairs with a diameter below the specified HDD threshold. Conversely, the 'Negative predictive value' represents the proportion of non-AGA individuals (V0) whose hair diameters do not reach the 20% threshold for the specified HDD value; HDD, hair diameter diversity.

**Fig 1:**
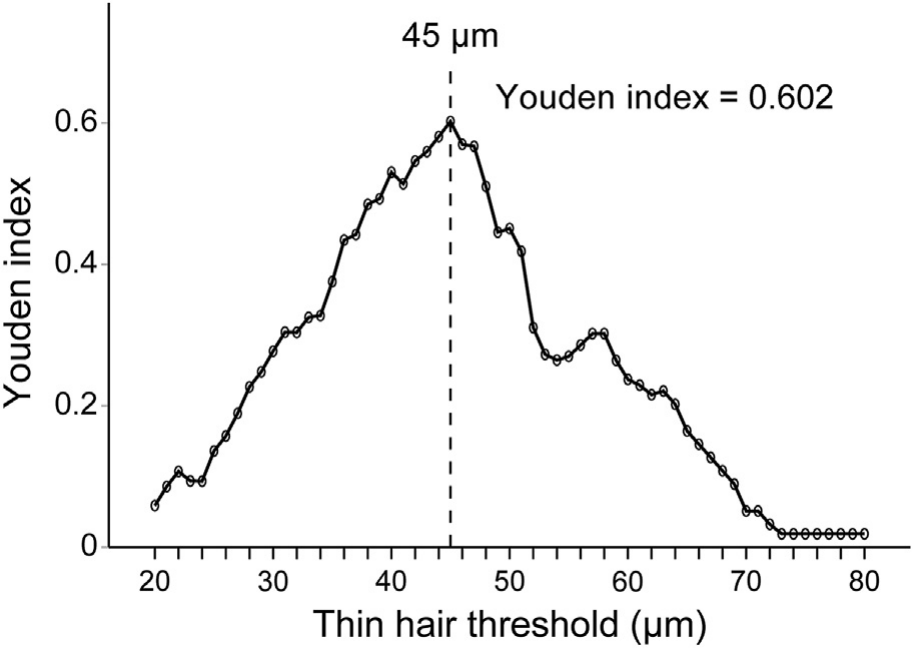
Optimal hair diameter threshold based on Youden index with a fixed cutoff at 20%. The *circles* indicate the Youden index values for different hair diameter thresholds, demonstrating the diagnostic efficacy at each point. The *curved line* connects these points, displaying the trend of diagnostic performance across hair diameter thresholds. The *dashed line* signifies the 45 μm threshold as the optimal point for diagnosis at a fixed cutoff of 20%.

Analysis of the Youden index to establish a diagnostic threshold for HDD was conducted by applying a fixed HDD criterion of 20%. It was revealed that a hair diameter threshold of 45 μm yielded the highest Youden index of 0.602 (Fig 1). Receiver operating characteristic curve analysis was utilized to assess the diagnostic accuracy of various hair diameter thresholds for AGA, with the highest AUC observed at the 45-μm threshold (Supplementary Figure 3, available via Mendeley at https://data.mendeley.com/datasets/f2zvbwjn3b/1). Further analysis as presented in Table IV represents the evaluation of hair diameter thresholds based on AUC, Youden index, and the optimal cutoff ratio for defining AGA. The AUC for the 30-μm threshold, a classic criterion for vellus hairs, was 0.770 with a CI of [0.703, 0.838] and a Youden index of 0.480, indicating a lower diagnostic capability. The 40-μm threshold had an AUC of 0.858 (CI [0.806, 0.919]) and a Youden index of 0.594. For the 45-μm threshold, the highest AUC of 0.884 (CI [0.840, 0.929]) and the highest Youden index of 0.659 were observed, suggesting the greatest diagnostic accuracy. The 50-μm threshold showed an AUC of 0.871 (CI [0.824, 0.918]) and a Youden index of 0.587. The optimal cutoff ratio for defining AGA increased with the threshold, being 11.48% for 30 μm, 15.28% for 40 μm, 21.65% for 45 μm, and 30.28% for 50 μm, respectively (Table IV). Even when analyzed separately, both the retrospective and prospective cohorts consistently showed the 45-μm threshold to possess the highest AUC and Youden index values, with the optimal cutoff ratio maintained at 21% (Supplementary Table I, available via Mendeley https://data.mendeley.com/datasets/f2zvbwjn3b/1). Therefore, when applying the 45-μm threshold as the optimal diagnostic criterion for HDD in the Korean population, the corresponding HDD threshold was established at 21.65%. This finding closely aligns with the previously established HDD standard of 20%, indicating its relevance and applicability.^6^

**Table IV:** Evaluation of hair diameter thresholds for androgenetic alopecia diagnosis based on area under the curve, Youden index, and optimal cutoff point

Thresholds	AUC		Youden index	Optimal cutoff ratio for defining AGA
	Value	CI	Value	Percentage
30 μm	0.770	[0.703, 0.838]	0.480	11.48
40 μm	0.858	[0.806, 0.919]	0.594	15.28
45 μm	**0.884**	[0.840, 0.929]	**0.659**	**21.65**
50 μm	0.871	[0.824, 0.918]	0.587	30.28

*AGA*, Androgenetic alopecia; *AUC*, area under the curve.

Bold means the highest value for AUC and Youden index.

## Discussion

Our study introduces a novel approach for the quantitative validation of the HDD standard in diagnosing AGA, with a specific focus on disease progression in the vertex region. The concept of HDD ≥20% as an objective tool for AGA assessment was initially introduced using a method similar to the phototrichogram, employing macrophotographs for hair analysis.^6^ However, the original definition of HDD ≥20% was not explicitly detailed, leading to an interpretation in subsequent studies that it involved cases where more than 20% of hairs are less than 40 μm in diameter.^2^^,^^4^^,^^20^ Our in-depth evaluation at a fixed 20% HDD cutoff revealed that a 45-μm threshold offers superior diagnostic performance for the Korean population, marked by the highest Youden index. This is particularly relevant considering that Korean individuals typically have thicker hair compared to those of Caucasian ethnicity.^14^, ^15^, ^16^, ^17^, ^18^, ^19^ Additional analysis, not limited to the fixed HDD cutoff, involving receiver operating characteristic curve evaluation for various hair diameter thresholds, indicated that the 45-μm threshold not only provides the highest AUC of 0.884, suggesting enhanced diagnostic accuracy for AGA, but also attains a peak Youden index at an optimal cutoff ratio of 21.65%. This finding closely aligns with the conventional 20% threshold, underscoring that the established HDD ≥20% remains a valid benchmark even in a different ethnicity. While the quantitative approach used in our study is intricate, it is noteworthy that other research has successfully utilized phototrichogram and image analysis software for measuring hair diameter.^21^ This precedent, coupled with the rapid advancements in artificial intelligence–driven imaging technology, holds promise for enhancing the precision and practicality of diagnostic methods in AGA in the near future.

However, this study has its limitations. Focusing exclusively on Korean males limits the generalizability of our findings to other ethnic groups or to females, who may exhibit different hair characteristics. Additionally, the absence of baseline HDD data for a nonalopecia group, beyond our V0 control group, restricts our understanding of HDD variations in a healthy population, a factor important for comparative analysis. While our sample size was sufficient, a larger cohort would provide more robust data and enable in-depth subgroup analyses. The cross-sectional nature of our study may not capture the progressive nature of AGA or changes in hair characteristics over time. Furthermore, using a specific hair diameter threshold may not account for individual variations in hair thickness, highlighting the need for a more personalized diagnostic approach. Diagnostic assessments for AGA often rely on established clinical scales like the Norwood-Hamilton, Ludwig, and BASP classifications,^2^^,^^6^^,^^22^ which, despite their ease of use and intuitive nature, are limited by their subjective quantification of the condition.^19^ Future research should explore the correlation between physicians' perceptions of HDD and its quantitative measurements to address these subjective aspects.

Nonetheless, our study quantitatively validates the conventional HDD 20% threshold through a sophisticated approach. We also have established a 45 μm hair diameter threshold, surpassing the conventional 40 μm standard, and better suited for the Korean population. In conclusion, adopting the HDD 20% continues to be a pivotal and practical method for early identification of vertex AGA. Furthermore, it is important to emphasize that the definition of thin hair should be tailored to specific ethnicities.

## Conflicts of interest

None disclosed.
